# Whole-Genome Sequences of 94 Environmental Isolates of *Bacillus cereus Sensu Lato*

**DOI:** 10.1128/genomeA.00380-13

**Published:** 2013-10-03

**Authors:** Géraldine A. Van der Auwera, Michael Feldgarden, Roberto Kolter, Jacques Mahillon

**Affiliations:** Laboratory of Food & Environmental Microbiology, Université Catholique de Louvain, Louvain-la-Neuve, Belgiuma; Broad Institute of Harvard and MIT, Cambridge, Massachusetts, USAb; Kolter Laboratory, Harvard Medical School, Boston, Massachusetts, USAc

## Abstract

*Bacillus cereus sensu lato* is a species complex that includes the anthrax pathogen *Bacillus anthracis* and other bacterial species of medical, industrial, and ecological importance. Their phenotypes of interest are typically linked to large plasmids that are closely related to the anthrax plasmids pXO1 and pXO2. Here, we present the draft genome sequences of 94 isolates of *B. cereus sensu lato*, which were chosen for their plasmid content and environmental origins.

## GENOME ANNOUNCEMENT

*Bacillus cereus sensu lato* is a species complex that groups six classically described species of ubiquitous Gram-positive spore-forming bacteria, including the eponymous *B. cereus sensu stricto*, the entomopathogen *Bacillus thuringiensis*, the rhizoid-looking *Bacillus mycoides* and *Bacillus pseudomycoides*, and *Bacillus anthracis*, the etiological agent of anthrax.

The members of this group were originally distinguished on the basis of their phenotypic differences, but over the past decade, advances in the understanding of the phylogenomics of this group have largely invalidated this classification. Instead, the members of the *B. cereus sensu lato* group are more appropriately viewed as forming one single species from which different ecotypes and pathotypes emerge in a dynamic fashion, leading in some cases to the formation of clonal complexes with specific phenotypes (1–6).

Many phenotypic properties that are specific to these ecotypes and pathotypes are directly related to the presence or absence of large plasmids that carry genes associated with those phenotypes. In the case of *B. anthracis*, the virulence plasmids pXO1 (192 kb) and pXO2 (96 kb) carry the anthrax toxin and capsule genes, respectively, as well as the associated regulatory elements (7). Furthermore, the large plasmids found in a number of previously sequenced *B. cereus sensu lato* strains of medical or industrial interest were observed to share a backbone with either the pXO1 or pXO2 anthrax plasmids. For example, in strains of *B. cereus sensu stricto* that are responsible for the *B. cereus*-associated emetic food poisoning syndrome, the genes encoding the emetic toxin cereulide are carried by a large plasmid that shares a common genetic backbone with the pXO1 anthrax plasmid (8, 9).

We have shown previously that select sequences of the shared pXO1 and pXO2 backbones can be found widely in environmental isolates of *B. cereus sensu lato* (10). We postulated that these are found in plasmids that are genetically related to the pXO1 and pXO2 plasmids (hence called pXO1-like and pXO2-like, respectively) and may play an important role in the ecotypic and pathotypic differentiation of *B. cereus sensu lato* organisms.

In order to gain deeper insight into the ecological distribution and genomic diversity of the pXO1-like and pXO2-like plasmids, we sequenced a panel of 94 isolates of *B. cereus sensu lato* organisms containing a variety of plasmids and having diverse environmental origins. This adds to the ~60 whole or draft genomes of *B. cereus* of various origins already available in GenBank.

*De novo* assemblies were generated from Illumina 101-base paired-end reads generated with two libraries, one from 180-bp fragments and one from 3-kb jumping libraries. The assemblies were constructed using AllPaths-LG (11). The protein-coding genes were predicted with Prodigal (12) and filtered to remove genes with ≥70% overlap to the tRNAs or rRNAs. The tRNAs were identified by tRNAscan-SE (13). The rRNA genes were predicted using RNAmmer (14). The gene product names were assigned based on top BLAST hits against the Swiss-Prot protein database (≥70% identity and ≥70% query coverage) and a protein family profile search against the TIGRfam HMMER equivalogs.

### Nucleotide sequence accession numbers.

All 94 draft genome sequences have been deposited at GenBank under the accession no. reported in Table 1 (10, 15–22).

**Table 1:** Strain characteristics

Sample source	Strain name	GenBank accession no.	Predicted plasmid(s)^*d*^	Reference
Soil, Greenland	VD048	AHEU01000000	pXO1	10
	VD078	AHEV01000000	pXO1	10
	VD045	AHET01000000	pXO2	10
	VDM022	AHFP01000000	pXO2	10
	VDM021	AHFU01000000	Neither	10
	VDM019	AHFO01000000	Neither	10
Soil, Spain	VD014	AHER01000000	pXO1	10
	VDM006	AHFT01000000	pXO2	10
	VDM034	AHFQ01000000	Neither	10
Soil, Scotland	VD142	AHCL01000000	pXO2	10
	VD148	AHFF01000000	pXO2	10
	VDM062	AHFS01000000	pXO2	10
	VD136	AHFC01000000	Neither	10
	VD140	AHFD01000000	Neither	10
	VD146	AHFE01000000	Neither	10
Water, Scotland	VD200	AHFM01000000	pXO1	10
	VD214	AHFN01000000	Neither	10
Soil, Martinique	VD133	AHFB01000000	pXO1	10
	VD131	AHFA01000000	Neither	10
Soil, Guadeloupe	VD107	AHEX01000000	pXO2	10
	VD115	AHEY01000000	pXO2	10
	VD102	AHEW01000000	Neither	10
	VD118	AHEZ01000000	Neither	10
Soil, Abu Dhabi, UAE	VD156	AHFH01000000	pXO1	10
	VD154	AHFG01000000	Neither	10
Soil, Dubai, UAE	VD169	AHFJ01000000	pXO1	10
	VD196	AHFL01000000	pXO1	10
	VD166	AHFI01000000	Neither	10
	VD184	AHFK01000000	Neither	10
Water, Belgium (small pond site)	VD021	AHES01000000	pXO2	10
	VD022	AHCK01000000	pXO1, pXO2	10
	VDM053	AHFR01000000	Neither	10
Soil, Belgium (site A)	HuA2-1	AHDV01000000	pXO1	10
	HuA2-3	AHDW01000000	Neither	10
	HuA2-4	AHDX01000000	pXO2	10
	HuA2-9	AHDY01000000	Neither	10
	HuA3-9	AHDZ01000000	pXO1	10
	HuA4-10	AHEA01000000	pXO2	10
Soil, Belgium (site B)	HuB1-1	AHEB01000000	Neither	10
	HuB2-9	AHED01000000	pXO2	10
	HuB4-4	AHEF01000000	Neither	10
	HuB4-10	AHEE01000000	pXO2	10
	HuB5-5	AHEG01000000	pXO2	10
	HuB13-1	AHEC01000000	Neither	10
Soil, Massachusetts (Boston site AG)	BAG1O-1	AHCN01000000	Neither	This study
	BAG1O-2	AHCO01000000	Neither	This study
	BAG1O-3	AHCP01000000	Neither	This study
	BAG1X1-1	AHCQ01000000	pXO1	This study
	BAG1X1-2	AHCR01000000	pXO1	This study
	BAG1X1-3	AHCS01000000	pXO1	This study
	BAG1X2-1	AHCT01000000	pXO1, pXO2	This study
	BAG1X2-2	AHCU01000000	pXO2	This study
	BAG1X2-3	AHCV01000000	pXO2	This study
	BAG2O-1	AHCW01000000	Neither	This study
	BAG2O-2	AHCX01000000	Neither	This study
	BAG2O-3	AHCY01000000	Neither	This study
	BAG2X1-1	AHCZ01000000	pXO1	This study
	BAG2X1-2	AHDA01000000	pXO1	This study
	BAG2X1-3	AHDB01000000	pXO1	This study
	BAG3O-1	AHFV01000000	Neither	This study
	BAG3O-2	AHDC01000000	Neither	This study
	BAG3X2-1	AHDD01000000	pXO2	This study
	BAG3X2-2	AHDE01000000	pXO2	This study
	BAG4O-1	AHDF01000000	Neither	This study
	BAG4X2-1	AHDH01000000	pXO2	This study
	BAG4X12-1	AHDG01000000	pXO1, pXO2	This study
	BAG5O-1	AHDI01000000	Neither	This study
	BAG5X1-1	AHDJ01000000	pXO1	This study
	BAG5X2-1	AHDL01000000	pXO2	This study
	BAG5X12-1	AHDK01000000	pXO1, pXO2	This study
Soil, Massachusetts (Boston site ES)	BAG6O-1	AHDM01000000	Neither	This study
	BAG6O-2	AHDN01000000	Neither	This study
	BAG6X1-1	AHDO01000000	pXO1	This study
	BAG6X1-2	AHDP01000000	pXO1	This study
Food	AND1407	AHCM01000000	pXO1	15
	K-5975c	AHEL01000000	pXO1	19
	TIAC219	AHCJ01000000	pXO1	20
	Schrouff	AHCI01000000	pXO1, pXO2	Mahillon et al., unpublished
	ISP3191	AHEK01000000	pXO1, pXO2	Dierick et al., unpublished
	ISP2954	AHEJ01000000	pXO1	Dierick et al., unpublished
Soil, China	B5-2	AHFW01000000	pXO1, pXO2	Sun et al., unpublished
Mammals, Poland	IS075	AHCH01000000	pXO1, pXO2	21
	IS195	AHEH01000000	pXO1, pXO2	21
	IS845/00	AHEI01000000	pXO1	21
Insect^*a*^	HD73	AHDU01000000	pXO2	22
Various origins^*b*^	MC67	AHEN01000000	Other	17
	MC118	AHEM01000000	Other	17
	MSX-A1	AHEO01000000	Other	16
	MSX-A12	AHEP01000000	Other	16
	MSX-D12	AHEQ01000000	Other	16
	BMG1.7	AHDQ01000000	Other	18
Various origins^*c*^	CER057	AHDS01000000		15
	CER074	AHDT01000000		15
	BtB2-4	AHDR01000000		15

a Reference strain for the pXO2-like plasmid pAW63.

b Strains predicted to carry other plasmids of interest.

c Strains predicted to carry plasmid fragments on the chromosome.

d Neither, neither pXO1 no pXO2; other, plasmids other than pXO1 or pXO2.
